# A study of biplanar crossed pin construct in the management of displaced pediatric supracondylar humeral fractures

**DOI:** 10.1007/s11832-014-0607-y

**Published:** 2014-09-03

**Authors:** Riazuddin Mohammed, Prabhudheer Bhogadi, Sreenivasulu Metikala

**Affiliations:** 1 Department of Trauma and Orthopaedics, Queens Hospital, Burton Upon Trent, DE13 0RB UK; 2 Department of Orthopaedics and Traumatology, Osmania Medical College and General Hospital, Hyderabad, Andhra Pradesh 500012 India; 3 Sri Venkateswara Orthopaedic Hospital, 100 & 1/101, George Reddy Street, Yerramukkapalli, Kadapa, Andhra Pradesh 516004 India

**Keywords:** Supracondylar humeral fracture, Biplanar crossed pins, Closed reduction, Percutaneous fixation, Technique

## Abstract

**Purpose:**

Study of a biplanar crossed pin construct by two crossed Kirschner wires in the management of displaced extension type supracondylar humeral fractures in children.

**Methods:**

Sixty-four patients with such fractures were included and treated according to the study protocol: after achieving closed reduction under general anesthesia with fluoroscopic control, two crossed Kirschner wires of equal diameter were inserted percutaneously. The first lateral wire tracts from the posterolateral corner of the epicondyle to the anteromedial cortex proximally. Then, the medial wire is inserted from the anteromedial corner of the epicondyle to the posterolateral cortex proximally after crossing the fracture site. Thus, a biplanar crossed pin construct was achieved, as each wire had two separate fixation points and the crossed construct was achieved not only in the coronal plane but also in the sagittal plane. Every effort was made to get this construct right at the very first attempt without repetition.

**Results:**

Two patients were lost to follow-up during the first postoperative year. The mean follow-up for the remaining 62 patients was 14.5 months (range 6–24 months). At the final follow-up, using Flynn’s overall modified classification, the clinical result was considered to be satisfactory in 60 (96.8 %) patients and unsatisfactory with poor result in two (3.2 %) patients. Technical error was thought to be the cause of the poor results. There were no postoperative neural or vascular complications.

**Conclusion:**

A biplanar crossed pin construct achieved by two Kirschner wires crossed in the coronal and sagittal planes is efficient to stabilize a displaced extension type supracondylar fracture of the humerus in children.

## Introduction

Extension type supracondylar humeral fracture is the most common injury around the elbow in the pediatric age group [1, 2]. Traditionally, this injury is classified based on the direction and degree of displacement, according to Gartland criteria [3, 4] (Table 1). Immobilization in a cast is generally accepted for an undisplaced fracture. The goal of treatment in a displaced fracture is to achieve and maintain a near anatomical reduction till the fracture shows signs of union. Closed reduction followed by percutaneous Kirschner wire fixation has become a standard method of treatment, together with a difference of opinion about the optimum pin configuration [5, 6, 8]. The two main options are crossed Kirschner wire fixation where one or more wires come from each of the epicondyles to cross above the fracture site, and parallel or divergent Kirschner wire fixation where two or more wires enter from the lateral epicondyle alone to stabilize the fracture. Biomechanical studies suggested the superiority of a crossed pin design over a parallel pin construct in terms of maintaining the fracture stability till healing occurred [7, 9–11, 23]. However, controversy persists regarding the number of Kirschner wires used in the treatment. The purposes of the present study are to describe the surgical technique along with the precautions needed during the procedure and also present the outcomes of a biplanar crossed pin construct achieved by two Kirschner wires in two planes for Gartland type III pediatric supracondylar humerus fractures.

**Table 1:** Modified Gartland classification for supracondylar humerus fractures

Type	Description
I	Undisplaced fracture
II	Displaced with intact posterior cortex
III	Completely displaced—either posteromedial IIIA or posterolateral IIIB
IV	Multidirectional instability with circumferential periosteal disruption

## Materials and methods

A prospective study was conducted among 64 consecutive children with Gartland type III extension type supracondylar humeral fractures at our institution from January 2008 to June 2013.

All children between 3 and 10 years of age and presentation within 5 days of injury were included in the study. The exclusion criteria were open fractures, associated neurological and/or vascular injury, fractures with multidirectional instability requiring open reduction, previous fracture in the same elbow, and associated ipsilateral fractures.

### Surgical technique and follow-up

After induction of general anesthesia, the child is positioned supine with the shoulder of the injured extremity close to the edge of the operating table with adequate supports. A single dose of parenteral cefuroxime as per the body weight of the child is administered at the time of induction of anesthesia. Closed reduction is performed under fluoroscopy, followed by antiseptic skin scrub and draping.

**Fig. 1:**
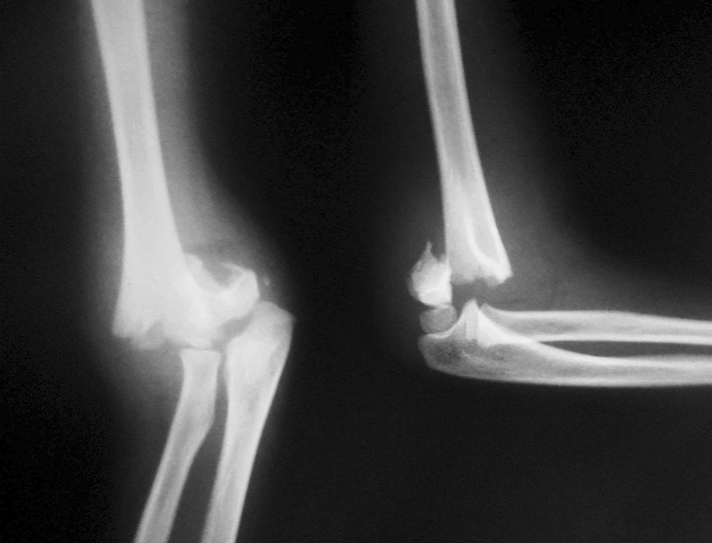
Radiograph of the right elbow in a 6-year-old boy: anteroposterior and lateral views showing displaced extension type supracondylar fracture of the humerus

**Fig. 2:**
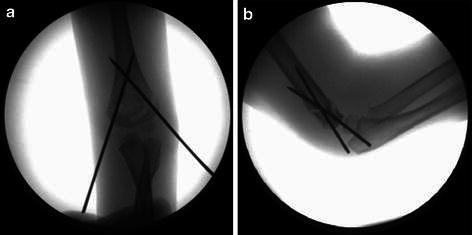
Fluoroscopy anteroposterior and lateral images showing two Kirschner wires, crossed in the coronal and sagittal planes

Two Kirschner wires of equal diameter are selected depending on the body weight of the child (1.5-mm diameter if the body weight is less than 15 kg, and 1.8 or 2 mm if over 15 kg). The first wire is passed from posterolateral corner of the lateral condyle, across the fracture site in the oblique direction, and proceeds proximally to gain the purchase in the anteromedial metaphyseal cortex. The second wire starts from the anteromedial corner of the medial epicondyle, crosses the lateral wire above the fracture site, and runs proximally to engage the posterolateral metaphyseal cortex. Thus, a biplanar crossed pin construct is achieved not only in the coronal plane but also in the sagittal plane (Figs. 1 and 2). Every effort is made to get this construct right at the very first attempt. Repeated attempts of pin insertion are avoided. The drilling pace of the power tool is reduced once the resistance of the opposite metaphyseal cortex is met and the secure purchase of the far cortex is achieved. Overshooting of the wires into the soft tissues is avoided. The injured elbow is kept in full flexion for lateral pin fixation and in 60°–70° of flexion for medial wire fixation.

Construct design and fixation stability is assessed in the anteroposterior (AP), lateral, and oblique fluoroscopy views with gentle stress in varus, valgus, internal, and external rotation. Both the wires are cut outside the skin without bending them, leaving the ends protruding about 2 cm. The limb is placed in a well-padded above-elbow back slab with the forearm in a neutral position and the elbow flexed around 60°–70°.

The children are discharged after overnight in-patient observation. The Kirschner wires and the back slab are removed after 3 weeks in the out-patient office after appropriate documentation of fracture healing (periosteal reaction and callus crossing the fracture site) is obtained. Active mobilization of the elbow is encouraged in the child soon after. All patients are evaluated at 3 weeks, 6 weeks, 3 months, and 6 months postoperatively. Clinical evaluation of range of elbow motion and carrying angle were measured using a goniometer. The system described by Flynn et al. [7, 16, 17] was used to assess the clinical results.

**Fig. 3:**
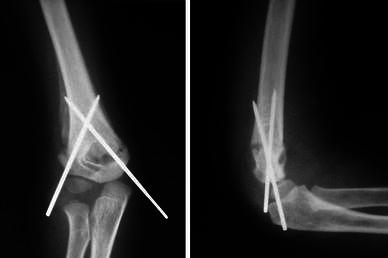
Follow-up radiograph at 3 weeks postsurgery showing fracture callus

**Fig. 4:**
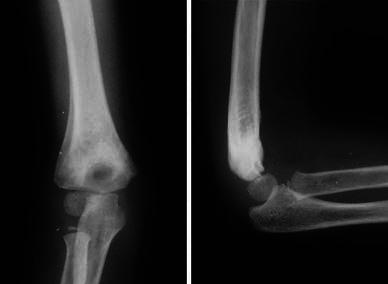
Fracture consolidation noted on the 3-month radiographs

**Fig. 5:**
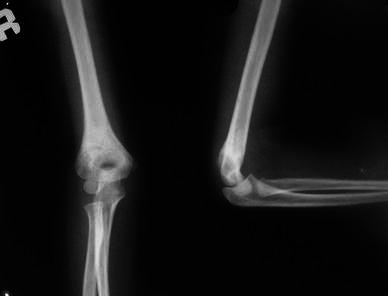
Final follow-up radiograph at 1 year depicting remodeling at the fracture site

Radiological assessment was made at the time of removal of wires and at final follow-up, on AP and lateral views of both elbows and frontal full views of both sides (Figs. 3, 4 and 5). The Baumann’s angle was recorded on the anteroposterior radiograph as described by Dodge [15].

## Results

Two patients were lost to follow-up during the first postoperative year. The mean follow-up for the remaining 62 patients was 14.5 months (range 6–24 months). There were 41 boys and 21 girls, with a mean age at presentation of 6.2 years (range 3.3–10.2 years). Forty-two fractures were dominant-sided injuries and the rest were on the non-dominant side. The mean interval between injury and operative procedure was 16.9 h (range 6–80 h). Anatomical reduction was achieved in all the cases. The mean time taken for radiological union was 3.2 weeks (range 2.5–3.8 weeks).

There were no postoperative neural or vascular complications. Two children had superficial pin-tract infection that was successfully treated with local dressings and a short course of oral antibiotics. None of the children had deep infection. Two patients had backed out lateral wire noted at their first follow-up (3 weeks postsurgery) and it was pulled out. The plaster slab was continued to be used for another week, after which the medial wire was removed.

**Fig. 6:**
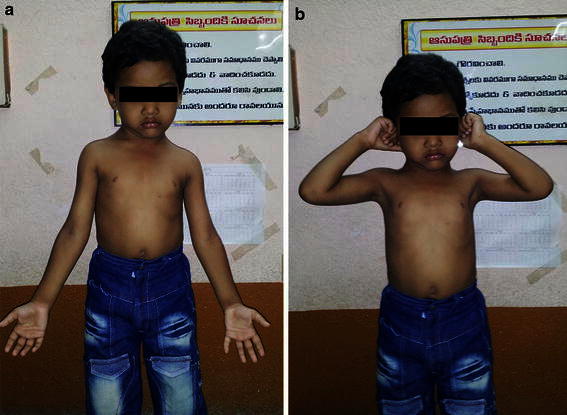
Clinical photographs showing good extension and flexion of the elbow at final follow-up

Three parameters were compared to the uninjured side and taken into account—Baumann angle (radiological), carrying angle (cosmetic), and range of motion (functional). The mean Baumann angle was 76.84° (range 70°–100°). At the final follow-up, 56 (90.3 %) patients had 0°–5° reduction of the carrying angle, 4 (6.5 %) had 6°–10° reduction, and 2 (3.2 %) had 20° and 25° reduction. Assessment of the elbow range of movement showed that 50 (80.6 %) patients lost less than 5° movement in the flexion–extension arc, 8 (13 %) lost 6°–10° of flexion, and 4 (6.5 %) lost 11°–15° of flexion (Fig. 6).

According to Flynn’s modified classification (Table 2), 60 (96.8 %) patients had a satisfactory result and 2 (3.2 %) had an unsatisfactory poor result at the final follow-up. The poor result in those two patients was due to backing out of the lateral wire, which happened owing to repeated attempts of Kirschner wire passage during surgery, resulting in poor bicortical purchase. The fracture in these two patients healed in varus collapse with an increase in the Baumann angle of 92° and 100°, and reduction in carrying angle of 20° and 25°, although the loss of functional movement was not more than 15° in both. The parents of both these patients were not keen on further surgical management, as the overall functional outcome was acceptable.

**Table 2:** Final outcome according to Flynn’s criteria and overall rating

Result	Overall rating	Cosmetic factor (carrying angle loss)	Functional factor (movement loss)
Satisfactory (*n* = 60)	Excellent	0°–5° (*n* = 56)	0°–5° (*n* = 50)
	Good	5°–10° (*n* = 4)	5°–10° (*n* = 8)
	Fair	10°–15° (none)	10°–15° (*n* = 4)
Unsatisfactory (*n* = 2)	Poor	>15° (*n* = 2)	>15° (none)

## Discussion

The treatment of displaced supracondylar fracture of the humerus in children by closed reduction and percutaneous pin fixation has consistently given satisfactory results [6, 7, 19]. A crossed pin construct in the form of a lateral and medial pin fixation method as popularized by Swenson [13] has the advantage of better biomechanical stability, although iatrogenic ulnar nerve injury is possible with the medial pin. Conversely, a lateral pinning entry method has the advantage of avoiding ulnar nerve injury, but this construct has been thought to be less stable. Biomechanical studies have shown that a medially and laterally crossed Kirschner wire configuration is 25 % more rigid than three lateral pins and 37 % stronger than two parallel lateral pins [11]. Redisplacement of the fracture has been reported to be a significant complication after using lateral Kirschner wires alone [9, 12]. A lateral Kirschner wire configuration may not allow full extension of the elbow, and, thus, prevents examination of the carrying angle during surgery [14].

Although many use crossed pin fixation, controversy persists regarding the number of Kirschner wires to be used for fixation. A greater number of wires inserted in each of the epicondyles may offer more stability, but multiple entry points over a small cartilaginous area would increase the chances of skin nipping, nerve entrapment, and pin-tract infections.

The standard crossed pin construct describes that each Kirschner wire should proceed from the epicondyles and cross proximal to the fracture site. This happens in the coronal plane alone but not in the sagittal plane, unless special attention is paid. We emphasize the technique of a biplanar crossed pin construct in which the medial and lateral Kirschner wires would achieve a crossed construct in the coronal as well as sagittal planes, providing adequate fracture stability. Though a biomechanical study of our pinning construct was not performed, a literature review mentions that crossed pinning of pediatric supracondylar fractures remains the most stable configuration [11, 23]. We believe that this could be extrapolated to assume that, when the wires cross in both the coronal and sagittal planes, the achieved construct would be more stable than in a uniplanar mode.

Ulnar nerve injury is a known complication after percutaneous medial–lateral crossed wire fixation methods. Hence, the lateral pinning technique [20], lateral cross-wiring technique [21], and antegrade nailing technique [22] were described to reduce iatrogenic ulnar nerve injury. However, there are standard techniques to prevent iatrogenic nerve palsy in a medial–lateral cross-pinning method, without even exposing the nerve. Initial swelling around the fractured elbow with displaced fracture fragments precludes palpation of the medial epicondyle and ulnar nerve. But once the fracture fragments are reduced under anesthesia and retained partly by the lateral wire, it is possible to feel the medial epicondyle if the elbow is kept in 60°–70° of flexion. Anatomically, the ulnar nerve rests in its native groove behind the medial epicondyle at this particular angle of elbow flexion and becomes prominent with increasing flexion [19]. Once the injured upper extremity is supported off the operating table and elbow flexion is maintained by the surgical assistant at this desired “safe angle”, the surgeon can comfortably hold the power drill with one hand and utilize the index finger along with the thumb of the other hand to feel the ulnar nerve and the anterior part of the medial epicondyle, respectively. Further, the medial wire is directed from an anteromedial to posterolateral fashion, thus avoiding stretching or tenting of the ulnar nerve in the postoperative period. This technique was employed in all 64 patients in this study and none had iatrogenic nerve injury. Rasool [18] reported that the safety of percutaneous cross-pinning seemed to be related to the surgeon’s experience.

Three children with neurological problems involving the median nerve were excluded from the study. Two of these presented with open fracture, with the proximal fragment projecting out through an anteromedial skin wound. They were dealt with by wound exploration, followed by internal fixation using two lateral wires. The third case was a 10-day-old closed fracture with multidirectional instability (Gartland type IV), which was managed by open reduction by a lateral approach and internal fixation using crossed Kirschner wires. Neurological recovery was noted in all children with these injuries in around 3 weeks’ time.

Careful attention during the drilling technique is of paramount importance. The drilling speed must be reduced immediately once the resistance of the opposite metaphyseal cortex is felt. The drilling is then continued at a low pace and stopped immediately at the point of appreciating loss of resistance to prevent overshooting of the wire. In this way, one can be sure of achieving a good purchase of the opposite cortex. Saline irrigation is done throughout the procedure to prevent thermal necrosis. Overprojecting into soft tissues would endanger the surrounding vital structures and pulling the wire back might compromise its purchase strength.

Enough emphasis was given in our series of patients to direct the wires in the correct direction at the very first attempt. Repeated attempts of wire passage would create false tracts, poor purchase with subsequent backing, and redisplacement in the postoperative period. This happened in two patients in whom the fracture healed in varus with an increase in the Baumann angle of >90°. The Kirschner wires are deliberately not bent before cutting and are left projecting outside the skin. We believe that the bending would create motion transmission and may weaken the purchase strength of the Kirschner wires. Despite this, no patient had internal wire migration in our series.

We also believe that the diameter of the wires is correctly chosen. For the children weighing less than 15 kg, 1.5-mm-diameter wires should suffice. If the weight of the child is more than 15 kg, the recommended diameter is either 1.8 or 2.0 mm. It is essential to ensure that both the Kirschner wires used are of equal diameter.

Table 3 shows the outcomes in relation to the timing of surgical intervention. The majority (90 %) of our patients were operated within a day of having sustained the injury. Our study numbers are not significant enough to make meaningful statistical correlation between time and intervention and final outcome. However, we believe that unnecessary delay is to be avoided in dealing with these injuries, as the soft tissue swelling worsens with passing time, making closed reduction difficult [24].

**Table 3:** Comparison between timing of surgery and final outcome

Interval from injury to surgery	Number of patients	Final outcome according to Flynn’s overall modified classification
<24 h	56	Satisfactory: 55
		Unsatisfactory: 1
24–48 h	4	Satisfactory: 3
		Unsatisfactory: 1
>48 h to day 4	2	Satisfactory: 2
		Unsatisfactory: 0

We have attempted to measure the angle between the K wires in AP and lateral projections, and analyzed the outcomes based on the angle of divergence (Table 4). It is not always possible to obtain “true” AP and lateral projections in all instances, and the angle measured between the K wires depends on the rotation of the humerus. Despite that, the angles between the K wires in the best possible true AP and lateral radiographs done in the postoperative period were measured.

**Table 4:** Angles measured between the K wires on plain radiographs and the associated outcomes

AP angle (°)	Lateral angle (°)	Number of patients	Complications	Flynn’s criteria outcomes
<50	<15	2	Lateral K wire backed out in both cases	Unsatisfactory, healed in varus collapse
	>15	4	Nil	Satisfactory
50–70	<15	11	Nil	Satisfactory, one lost to follow-up
	>15	12	Superficial infection in one patient	Satisfactory
>70	<15	16	Superficial infection in one patient	Satisfactory
	>15	19	Nil	Satisfactory, one lost to follow-up

Both the cases in which the lateral K wires backed out resulting in varus collapse of the fracture occurred when the angle of divergence of the wires was the lowest. We believe that the angle between the K wires should be as divergent as possible in order to achieve greater biomechanical stability.

A limitation of our study is the lack of direct comparison with other forms of reduction and fixation. However, our series has a reasonable number of subjects with good follow-up, with prospectively collected data and satisfactory outcomes to its merit. In the future, further larger series and biomechanical comparison studies may validate the study.

## Conclusions

On the basis of our results, we conclude that a biplanar crossed pin construct achieved by two Kirschner wires, crossed in two planes, is efficient for stabilizing a displaced extension type supracondylar humeral fracture in children and provided a safe and effective surgical technique.
